# Imaging and quantitative analysis of insecticide in mosquito net fibers using Time-of-Flight Secondary Ion Mass Spectrometry (ToF-SIMS)

**DOI:** 10.1371/journal.pone.0209119

**Published:** 2018-12-26

**Authors:** Stephen C. Smith, Chuanzhen Zhou, Fred A. Stevie, Roberto Garcia

**Affiliations:** 1 Division of Parasitic Diseases and Malaria, Centers for Disease Control and Prevention, Atlanta, Georgia, United States of America; 2 Analytical Instrumentation Facility, North Carolina State University, Raleigh, North Carolina, United States of America; Brandeis University, UNITED STATES

## Abstract

Time-of-flight secondary ion mass spectrometry (ToF-SIMS) analysis was used to qualitatively and quantitatively assess the distribution of permethrin insecticide on the surfaces and interiors of Olyset long-lasting insecticidal net (LLIN) fibers. Total insecticide content in LLINs has been established using many analytical methods. However, it is important to quantify the bioavailable portion residing on the fiber surfaces for incorporated LLINs. ToF-SIMS is a very surface sensitive technique and can directly image the spatial distribution of permethrin insecticide on the surface of Olyset fibers. Surface permethrin appeared as patchy deposits which were easily removed by acetone and reappeared after several days as interior permethrin migrated (bloomed) from the fiber interior. After a wash/incubation cycle, permethrin deposits were more diffuse and less concentrated than those on the as-received fibers. ToF-SIMS is particularly sensitive to detect the Cl^-^ ion, which is the characteristic ion of permethrin. Ion implantation and quantification of dopants using SIMS is well established in the semiconductor industry. In this study, quantitative depth profiling was carried out using ^35^Cl^-^ ion implantation to correlate secondary ion yield with permethrin concentration, yielding a limit of detection of 0.051 wt% for permethrin. In some cases, surface concentration differed greatly from the fiber interior (>1 μm below the surface). Two- and three-dimensional mapping of Cl at sub-micrometer resolution showed permethrin to be dissolved throughout the fiber, with about 2 vol% residing in disperse, high-concentration domains. This suggests that these fibers fall into the class of monolithic sustained-release devices. It is expected that ToF-SIMS can be a valuable tool to provide insight into the insecticide release behavior of other LLIN products, both current and future.

## Introduction

Insecticide-treated mosquito netting has been a mainstay of malaria control programs worldwide for more than 15 years. From 2004 to 2014, more than one billion long-lasting insecticidal nets (LLINs) in the form of bednets have been delivered into malaria-endemic regions [1] and are considered the largest factor in the prevention of approximately 6.2 million malaria deaths since 2001 [2]. As of June 2017, 19 LLIN products, made by 14 manufacturers, have received either interim or full recommendation by the World Health Organization Pesticide Evaluation Scheme (WHOPES). The insecticides used in these products are limited to permethrin, deltamethrin, alphacypermethrin, and chlorfenapyr, chosen for their low mammalian toxicity, low volatility, and high contact-toxicity against mosquitoes [3].

Two approaches are used to manufacture LLINs. One is to topically apply a durable insecticide formulation onto netting made with multifilament polyester yarn. The alternative is to mix insecticide into a polymer, either high-density polyethylene (HDPE) or polypropylene (PP), melt-spin the polymer into fibers and then convert these fibers into netting. This latter approach, insecticide “incorporation,” is intended to produce nets having insecticide present throughout the bulk of the fiber. In principle, the fiber acts as a sustained-release device, presenting a portion of the insecticide on its surface where it can interact with an alighting mosquito. If the insecticide is stripped from the fiber surface by e.g., washing or abrasion, it is replenished by spontaneous migration of additional insecticide from the interior [4–6]. As of June 2017, 12 out of 19 WHOPES-recommended LLINs utilize the incorporation approach.

Analytical methods for measuring the total insecticide content in LLINs have been established [7–9], but for incorporated LLINs it is also important to quantify the bioavailable portion residing on the fiber surfaces. To some extent, this can be measured using mosquito bioassays, in which mosquitoes are directly exposed to net specimens to assess their insecticidal potency, with the results expressed as the percentage of mosquitoes incapacitated and killed.[10] These bioassays require large numbers of suitable mosquitoes in order to yield statistically valid results, and therefore require the availability of mosquito rearing and handling facilities. Even under the best conditions, results can be difficult to reproduce, largely because of the inherent variability of the mosquitoes themselves. Furthermore, mean mosquito mortality rises nonlinearly with increasing surface insecticide concentration, making it an imprecise measure of insecticide surface concentration and blooming dynamics.

A suitable chemical assay for insecticide on fiber surfaces has been elusive. Tami *et al* [11] described washing permethrin-incorporated net samples for 1 minute in acetone followed by gas chromatographic analysis of the wash solution. However, the results showed no correlation with bioassay data. For example, samples measuring 189, 197, and 324 mg of surface permethrin per kg of net elicited 74, 22, and 0% effective mortality, respectively, in bioassays. Inconsistent removal of the insecticide during the acetone wash may be one reason for this lack of correlation. Another reason may be that the surface concentrations may have changed during the time between the bioassays and the chemical analyses, which were conducted in separate laboratories in different countries.

Azondekon *et al* [12] described a controlled rub-off method using lens paper to collect insecticide from the surfaces of permethrin-incorporated nets. The papers were then extracted with acetone and the extract analyzed for permethrin using gas chromatography. It was found that nets that had >2.73 μm per rub-off elicited >80% mortality in bioassays with 75.9% specificity and 87.0% sensitivity. This method is intended to be used as a pass/fail test, and has not been validated as a means to quantify permethrin residing on the fiber surfaces.

Lacking in these investigations has been a method to directly observe and quantify insecticide on the surfaces of LLIN fibers. Such capability would serve to confirm that a given assay procedure for removing surface insecticide (e.g., solvent washing or controlled rubbing) achieves complete removal without disturbing the subsurface concentration. It would also help characterize the rate and extent of insecticide migration from the subsurface to surface during regeneration. Furthermore, it could lead to a more thorough understanding of the dynamics of incorporated-insecticide nets, assisting in the development of improved products in the future.

The present study examines the use of time-of-flight secondary ion mass spectrometry (ToF-SIMS) to probe insecticide on the surfaces and interiors of LLIN fibers. During ToF-SIMS analysis, a sharply focused ion beam is used to sputter molecules from the top 1–2 monolayers of a sample, creating (secondary) ions that are then directed into a mass spectrometer for chemical species identification and quantification. The incident ion beam is typically focused to a diameter of 0.3 μm, and the total area investigated is correspondingly small, typically 50 μm × 50 μm. If the beam is moved laterally stepwise, the specimen can be scanned to create a two-dimensional map of chemical species at sub-micrometer resolution. This analysis can be carried out on a fiber surface or on the cross-section of a cut fiber. Furthermore, a second ion beam can be used to sputter off successive layers (1–2 nm each) of the sample, allowing direct analysis of the underlying material, leading to a three-dimensional depth profile.

Insecticide-incorporated LLIN fibers were expected to be compatible with ToF-SIMS analysis for several reasons. First, the area of spot analysis can be as small as 300 nm, which is up to 700 times smaller than the 120–200 μm diameter of an individual fiber, thus making two-dimensional surface chemical mapping and depth profiling conceivable. Second, the insecticides of interest are halogenated, and would be expected to generate high yields of either chlorine or bromine ions for detection. Furthermore, the insecticide concentration in fresh LLINs of 0.2–2 wt%, depending on the insecticide, is well within the detectable range for halogenated compounds by ToF-SIMS. On the other hand, ToF-SIMS analysis of fiber surfaces presents some challenges. The rounded fiber surfaces cause spectral peak broadening, reducing mass resolution, due to differential travel times of secondary ions from the sample to the detector. In addition, the extremely small region of analysis requires that each specimen must be analyzed at multiple locations in order to ensure representative sampling.

**Fig 1:**
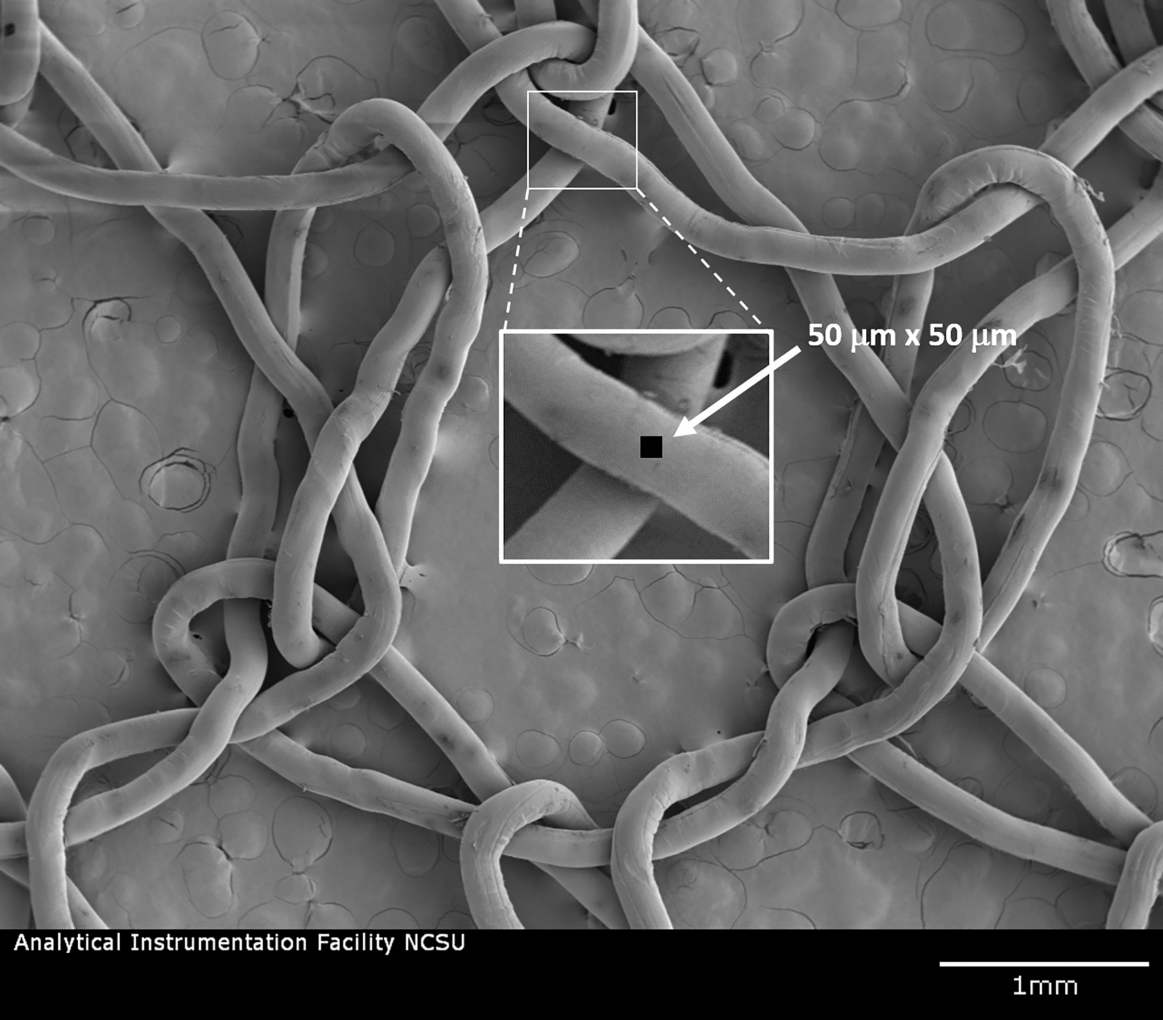
Scanning electron micrograph of Olyset netting illustrating the size of the fiber relative to a 50 μm x 50 μm analysis area. Image obtained at 5 kV after sputter coating the specimen with gold palladium.

**Fig 2:**
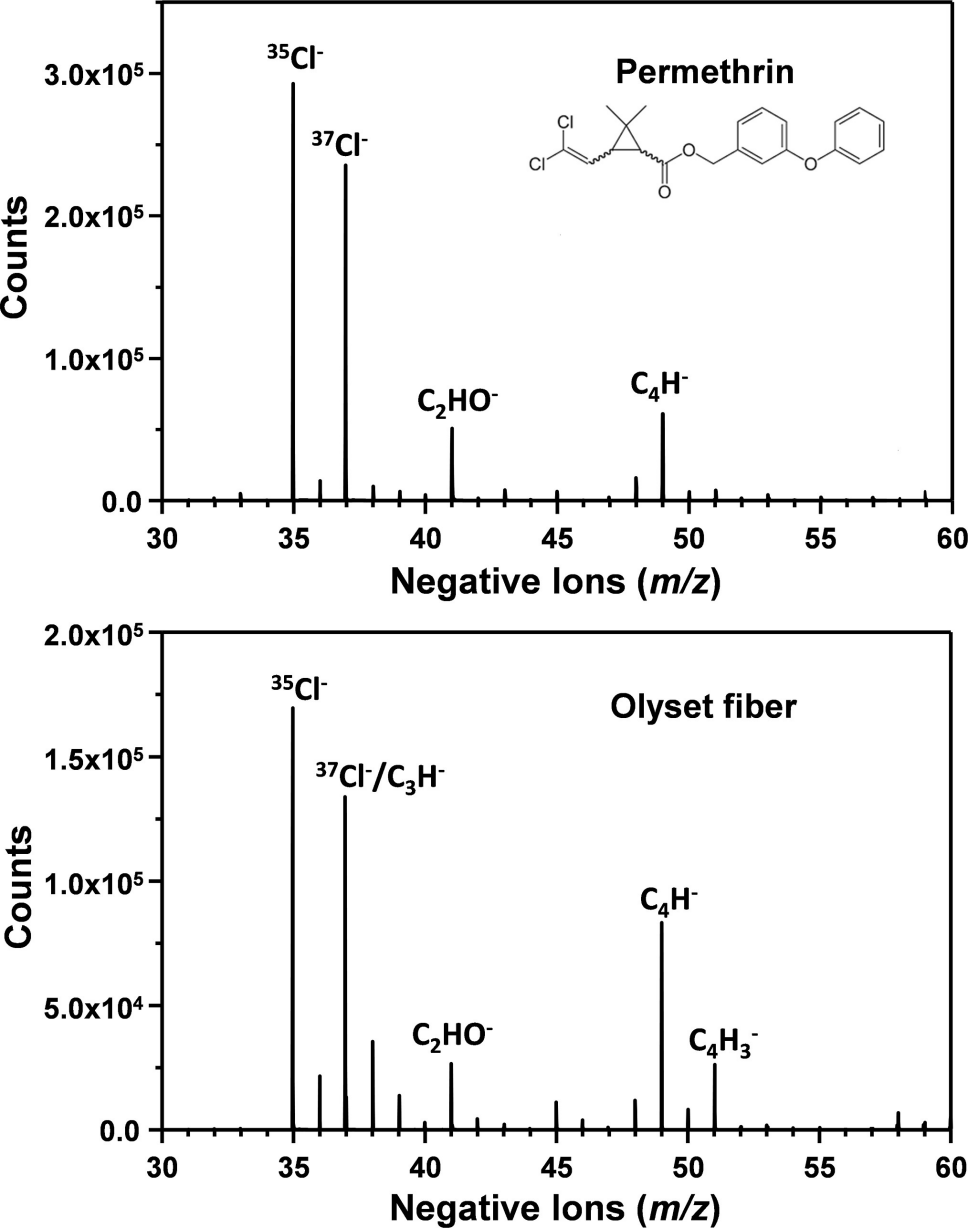
Negative secondary ion high resolution mass spectra of permethrin and an Olyset fiber surface acquired by ToF-SIMS. The strong peaks at m/z 35 and 37 correspond to the two naturally occurring stable isotopes of chlorine. The peak at m/z 37 may also include a contribution from C_3_H^-^ ion. Note that the ratio of ^35^Cl^-^ and ^37^Cl^-^ appears to be higher than their naturally abundant 3:1 ratio in the spectrum. For pristine permethrin, this is due to saturated ^35^Cl^-^ and ^37^Cl^-^ ion counts to the detector. On pure Permethrin and as received Olyset fiber, ^35^Cl saturation is observed. However, this signal saturation does not occur for washed and incubated samples because the permethrin concentration is significantly lower. For the Olyset fiber surface, the contribution of C_3_H^-^ at m/z 37 from HDPE is significant, but the instrument has sufficient mass resolution to separate ^37^Cl^-^ and C_3_H^-^. Separation of ^37^Cl^-^ and C_3_H^-^ cannot be shown on this plot, they are too close in mass. This plot is intended to show a wider mass range. However, if we zoom in at the m/z 37 region, we clearly see two individual peaks. The C_4_H_3_^-^ ion at *m/z* 51 was used in this study as a HDPE reference peak.

This report focuses on only one commercial LLIN product, Olyset Net (Sumitomo Chemical Co., Ltd., Tokyo, Japan). This product is a permethrin-incorporated HDPE monofilament net and was the first LLIN to be fully recommended by WHOPES [13]. It was selected for this study because the relatively high insecticide content (specified to be 2 wt%) and large fiber diameter (ca. 180 μm) were thought to make it particularly accessible to chemical mapping of 50 μm x 50 μm areas (Fig 1). Permethrin is a dichlorinated insecticide and mass spectral analysis confirmed the presence of a strong chlorine signal arising from both neat permethrin and the permethrin-incorporated net (Fig 2). It is expected that analysis methods developed using Olyset can be readily applied to other insecticide-incorporated LLINs.

ToF-SIMS has been used previously to observe the distribution of low molecular weight additives in polymer matrices [14–18]. While it is relatively straightforward to collect qualitative information on the morphology and distribution of the additive under investigation, quantitative measurements can be challenging. The intensity of the analyte signal is strongly matrix-dependent, which necessitates the development of case-specific calibration curves. This can be done by preparing a set of reference specimens with known analyte concentrations in an identical matrix, i.e. external calibration standards. But in many situations it is not possible to create such standards, especially if the sample under study was prepared using proprietary processes and ingredients. Such is the case for LLINs.

For this study, a quantification method was developed [19] using polyethylene samples implanted with a known dose of ^35^Cl^-^, followed by depth profile analysis to permit calculation of the relationship between ^35^Cl concentration and signal intensity (the relative sensitivity factor, or RSF). Using the RSF, the ^35^Cl^-^ signal in a test specimen can be directly converted to concentration, thereby permitting the ^35^Cl concentration to be depth-profiled. Assuming all of the ^35^Cl in the test sample arises from permethrin molecules, the permethrin depth profile can be calculated.

## Materials and methods

Olyset net samples were obtained from Sumitomo Chemical Co. Ltd. (Tokyo, Japan).

ToF-SIMS analysis was performed using a TOF.SIMS 5 (IONTOF, Münster, Germany) instrument. A 25 keV, 0.4 pA Bi_3_^+^ analytical beam was used for imaging and quantitative analysis, while a 3 keV, 20 nA Cs^+^ beam was used to sputter the sample for depth profile analysis. A 45° angle of incidence was used for both beams. For depth profiling, a 120 μm x 120 μm crater was sputtered and analysis was carried out on a 50 μm x 50 μm area at the center of the crater. Typical mass resolution for the analyses was 4000 at 29 *m/z* with high current bunched mode to obtain high mass resolution spectra. This resolution was sufficient to resolve any mass interference between the ^35^Cl^-^ and ^37^Cl^-^ peaks. Crater depths were measured using a Dektak 150 profilometer (Veeco Instruments, Inc., Oyster Bay, NY) and a Keyence VKX 250 optical profiler (Keyence Corp., Itasca, IL). Samples were analyzed as received and after pressing using a Carver hydraulic press at approximately 7000 kPa (1000psi) Carver press. Incubation was conducted with a Fisher Scientific Isotemp Incubator model 11-690-637F (6842). Typical temperature settings were 30–33°C.

Calibration for quantitative analysis was carried out using a ^35^Cl^-^ ion implantation method described previously [19] with a dose of 5x10^15^ atoms/cm^2^ at 140 keV. Implantation services were provided by Leonard Kroko, Inc. (Tustin, CA). Implantation was made simultaneously into a silicon control, HDPE (at two different thicknesses), and pressed Olyset fibers. From depth profile analysis the relative sensitivity factor (RSF) for ^35^Cl^-^ secondary ions was found to be 2.2x10^16^ atoms/cm^3^ per count. Assuming that the RSF of ^35^Cl^-^ and ^37^Cl^-^ are identical, the limit of detection (LOD) for Cl in HDPE samples was found to be 1.5x10^18^ atoms/cm^3^, which corresponds to an LOD for permethrin of 0.051 wt%. This LOD was considered sufficiently low for the study of these nets, which were expected to have overall permethrin contents ranging from zero to 2.5 wt% permethrin.

Quantification of these results was challenging because the rounded and somewhat rough surface topography of the fibers caused secondary ions of a given *m/z* to arrive at the time-of-flight analyzer at a range of times. This is manifested as peak-broadening and reduced mass resolution and increases the risk of interference between neighboring peaks. To reduce this effect, samples were pressed in a hydraulic press with sufficient pressure to slightly flatten the fibers (ca. 7000 kPa). Also, since ToF-SIMS collects data primarily from the top nanometer of the surface, surface contamination of the species of interest can alter the results. Therefore, gentle sputtering on the area of analysis using an unpulsed Bi^+^ beam was conducted with spectra acquired on the same area before and after sputtering.

**Fig 3:**
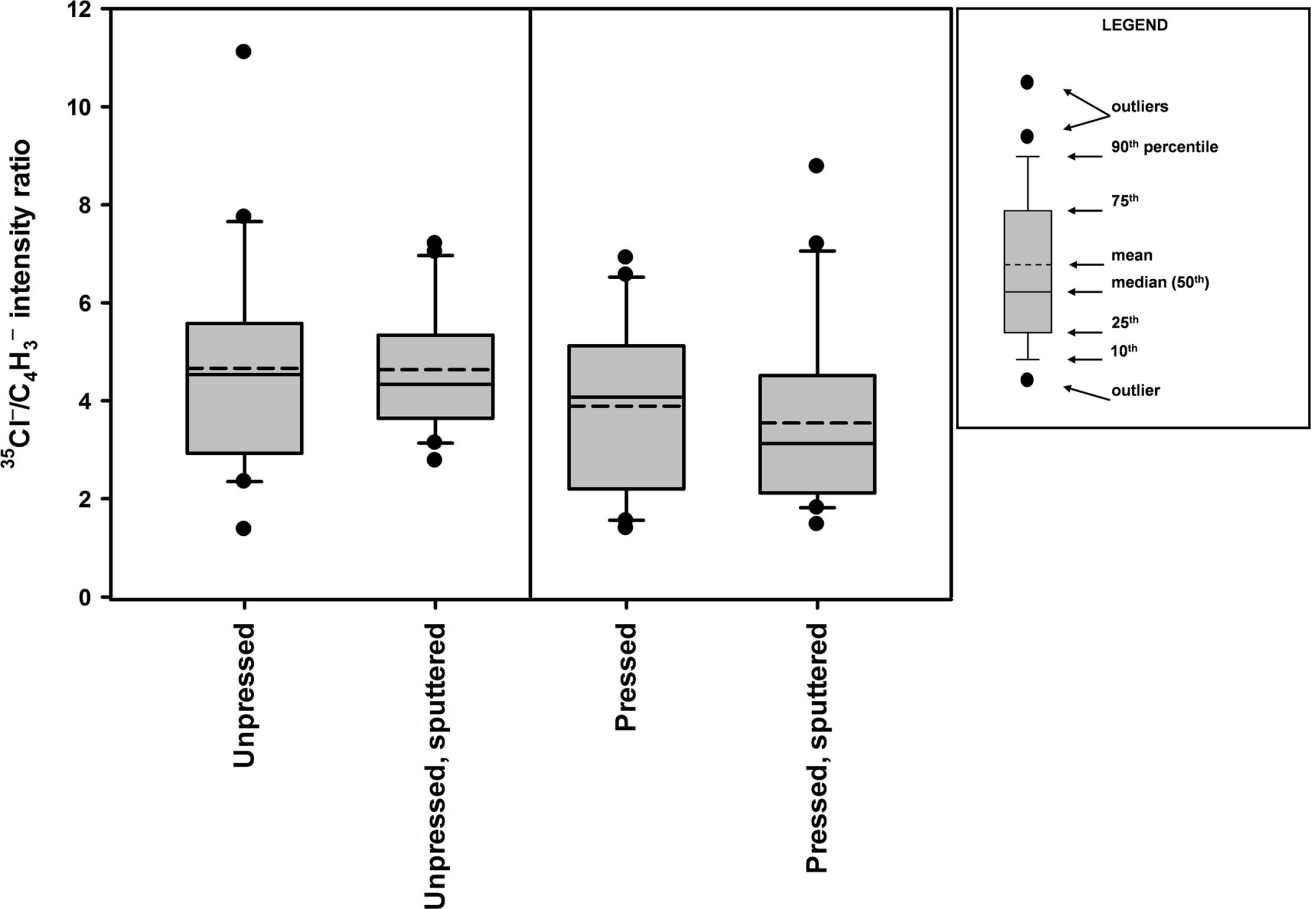
Box plots showing the effects of sample pressing and gentle sputtering on the ^35^Cl^-^/C_4_H_3_^-^ secondary ion intensity ratios from Olyset fibers obtained from ToF-SIMS negative ion high resolution mass spectra. Each plot represents readings from 20 random locations on a single sample specimen. Analysis area of each location is 500 μm × 500 μm.

To test the effects of sample pressing and sputtering on the secondary ion emissions, measurements were made on 20 random locations on an Olyset net specimen as received (i.e., unpressed and unsputtered), and on a specimen obtained from pressed adjacent area. Afterwards, both specimens were sputtered and measurements were repeated. The results (Table 1 and Fig 3) show a wide range of ^35^Cl^-^/C_4_H_3_^-^ intensity ratios, depending on the point of analysis, indicating extremely non-uniform distribution of permethrin on the fiber surface. The 95% confidence intervals for the mean for each of the samples overlap, suggesting that the processes of pressing and sputtering do not interfere significantly with the results. All subsequent analyses were made with specimens that were pressed and lightly sputtered.

**Table 1:** Statistical summary of ^35^Cl^-^/C_4_H3- secondary ion ratios for Olyset net as-received (unpressed), and pressed, with and without subsequent sputtering.

	Unpressed	Unpressed, sputtered	Pressed	Pressed, sputtered
Number of locations analyzed (N)	20	20	20	20
Median	4.53	4.34	4.07	3.13
Mean	4.66	4.63	3.89	3.55
Standard Deviation	2.22	1.28	1.66	1.94
Relative Standard Deviation (%)	47.6	27.7	42.8	54.7
95% Confidence Interval for Mean	3.69–5.63	4.07–5.20	3.16–4.62	2.70–4.40

## Results

### ToF-SIMS imaging of permethrin on Olyset fiber surfaces

**Fig 4:**
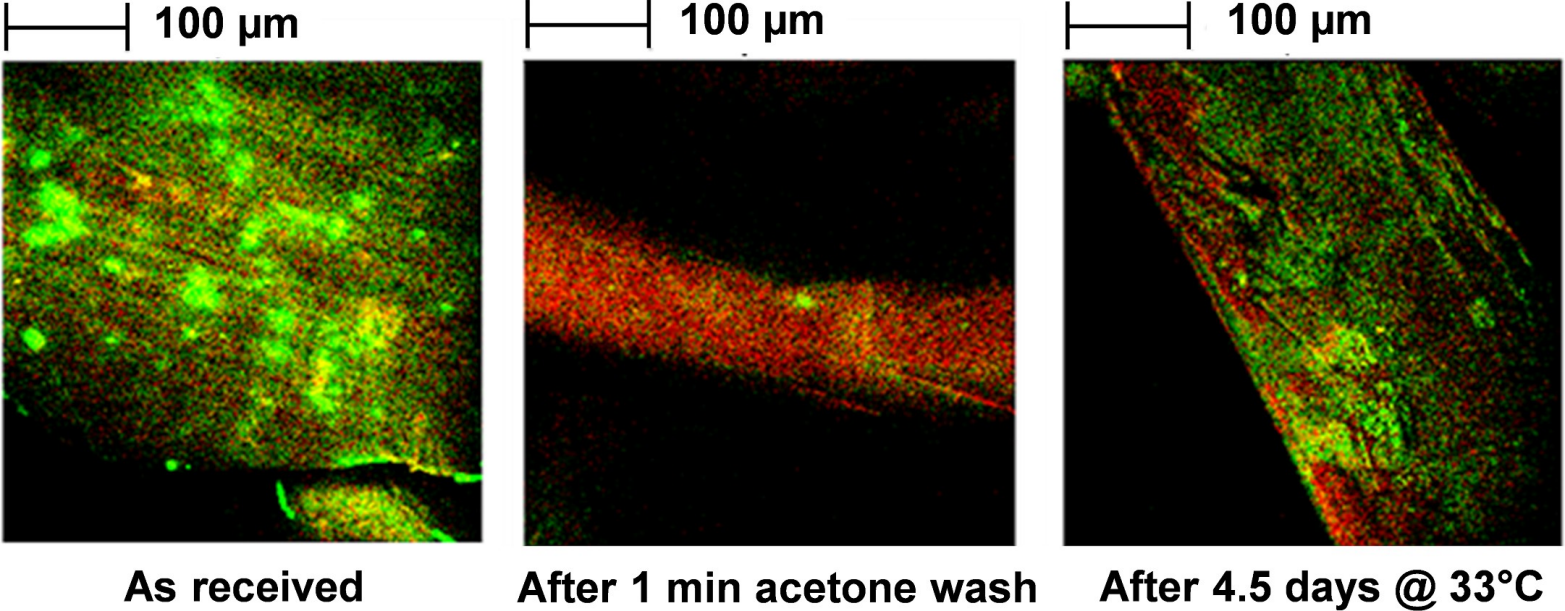
ToF-SIMS negative ion high spatial resolution images showing ^35^Cl^-^ distribution on Olyset fiber surfaces as received, after acetone wash, and after incubation for 4.5 days at 33°C. Chlorine regions are shown in green. Analysis area of each location is 400 μm × 400 μm.

Fig 4 shows typical high lateral resolution images of secondary ion emissions from Olyset fiber surfaces. In the as-received sample, ^35^Cl^-^ emissions, presumably arising from permethrin, appear as bright discontinuous patches on the fiber surfaces. In contrast, the C_4_H_3_^-^ emission from HDPE appears uniform. With a sample washed in acetone for 1 min, almost no ^35^Cl^-^ emission is observed, consistent with the removal of the acetone-soluble permethrin from the fiber surface. After incubating an acetone-washed sample for 4.5 days at 33°C, ^35^Cl is again detectable on the fiber surface, as would be expected from the migration of permethrin from the subsurface. Notably, the regenerated ^35^Cl has a more uniform and diffuse appearance than was seen in the as-received sample. Longer incubation times than 4.5 days were tried but did not show significant change. The temperature was chosen to mimic reasonable ambient temperatures in sub-Saharan Africa. Redistribution of insecticide was noted after 33°C baking and the temperature was dropped slightly to 30°C for subsequent analyses.

**Fig 5:**
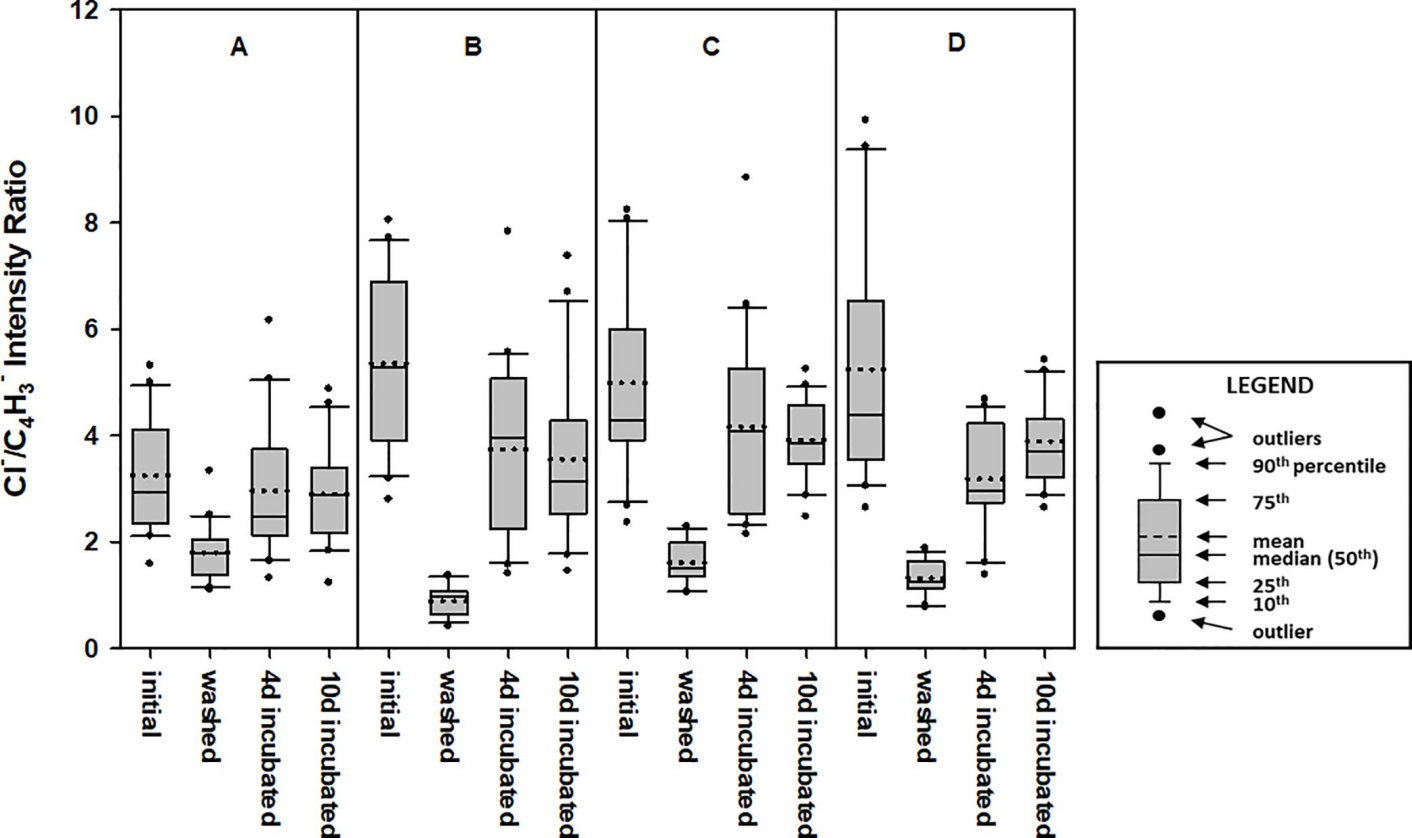
Box plots showing the regeneration of ^35^Cl^-^ at the surface of Olyset fibers from four specimens (A through D) after washing with acetone and subsequent incubation for 4 and 10 days at 30°C. Initial readings were carried out after samples were pressed and sputtered. Analysis area of each location is 500 μm × 500 μm.

Results for washed and regenerated samples are shown for four samples in Table 2 and Fig 5. Acetone washing reduced the relative ^35^Cl^-^ intensity on the fiber surfaces by 44.6–88.3%. Incomplete removal of detectable ^35^Cl may be due to (1) incomplete removal of permethrin from the surface, (2) complete removal of permethrin, but migration of permethrin from the subsurface during the time between washing and data collection, or (3) the presence of one or more additional chlorine-containing substances that are not acetone-soluble. Some combination of these factors may also be possible. The ToF-SIMS instrument has sufficient mass resolution to eliminate mass interference as a possibility.

**Table 2:** Statistical summary of ^35^Cl^-^/C_4_H_3_^-^ intensity ratios for four samples washed and incubated at 30°C.

Sample		N	Median	Mean	Standard Deviation	Relative Standard Deviation (%)	95% Confidence Interval for the Mean	% Change in Mean vs Initial
A	Initial	20	2.95	3.25	1.06	32.7	2.79–3.72	-
	Washed	20	1.80	1.80	0.53	29.4	1.57–2.04	-44.6
	4d incubated	20	2.47	2.97	1.26	42.5	2.42–3.52	-8.8
	10d incubated	20	2.88	2.91	0.92	31.7	2.50–3.31	-10.7
B	Initial	20	5.29	5.36	1.59	29.6	4.66–6.06	-
	Washed	20	0.97	0.89	0.30	33.5	0.76–1.03	-88.3
	4d incubated	20	3.96	3.75	1.70	45.3	3.00–4.49	-30.1
	10d incubated	20	3.14	3.56	1.53	43.0	2.89–4.23	-33.6
C	Initial	20	4.29	5.00	1.76	35.3	4.22–5.77	-
	Washed	10	1.50	1.61	0.38	23.8	1.37–1.85	-67.7
	4d incubated	20	4.09	4.17	1.70	40.9	3.42–4.91	-16.6
	10d incubated	20	3.86	3.92	0.71	18.1	3.61–4.23	-21.5
D	Initial	20	4.40	5.25	2.23	42.4	4.27–6.22	-
	Washed	20	1.27	1.32	0.34	25.9	1.17–1.47	-74.8
	4d incubated	20	2.97	3.19	1.01	31.8	2.75–3.64	-39.2
	10d incubated	20	3.72	3.90	0.81	20.7	3.54–4.25	-25.8

Incubating the washed samples at 30°C resulted in a substantial recovery of detectable ^35^Cl on the fibers, consistent with migration of permethrin from the subsurface to the surface. In all cases, recovery was partial with the mean ^35^Cl levels being -8.8% to -39.2% of the initial levels after 4 days incubation and -10.7% and -33.6% after 10 days. It is apparent from these data that the migration of permethrin to the fiber surfaces was essentially completed within 4 days at this temperature.

### Quantification and depth profile analysis

The results above show the relative levels of permethrin on the surfaces of Olyset fibers and the micrometer-scale lateral variability in these levels.

**Fig 6:**
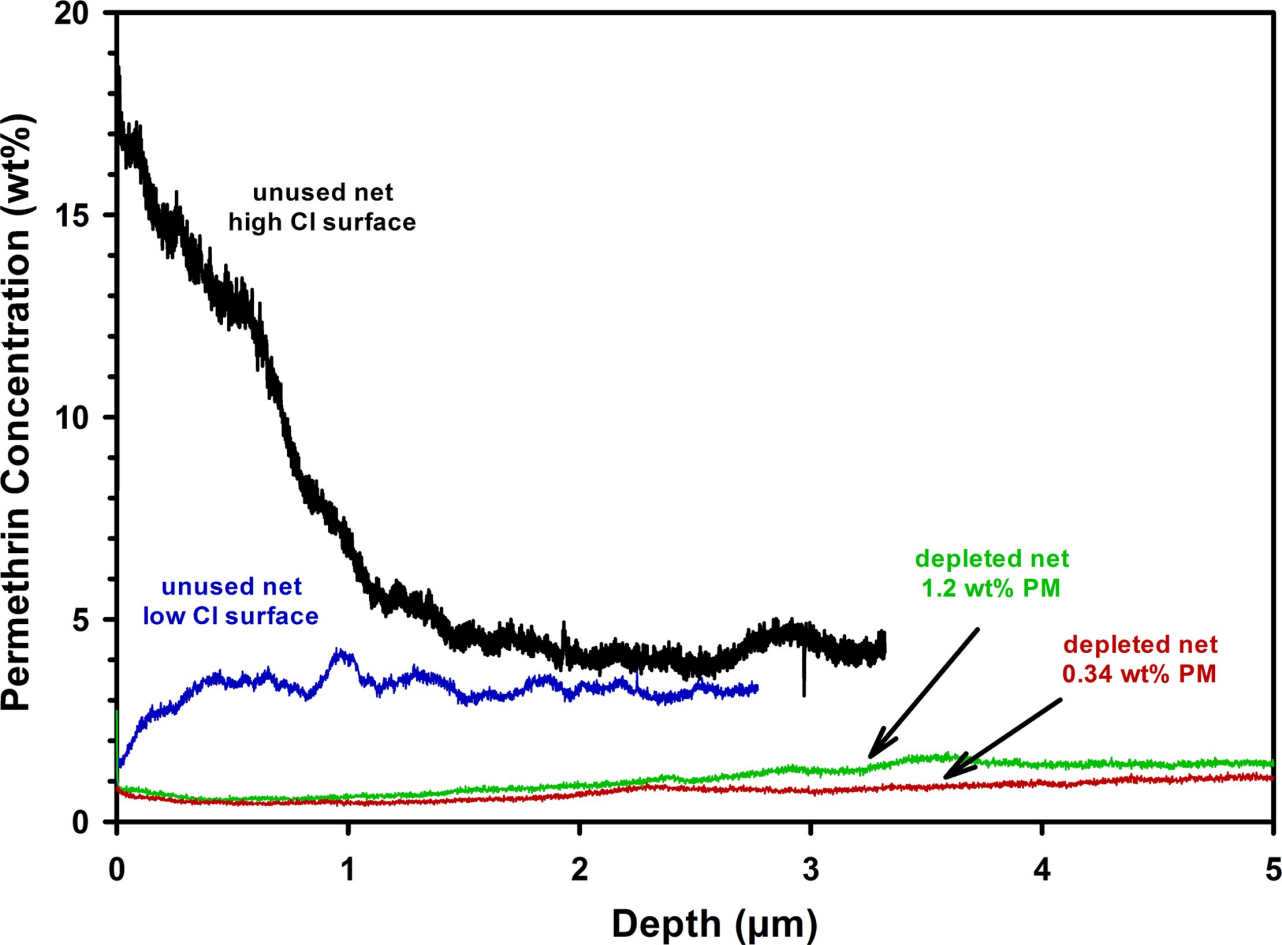
ToF-SIMS Depth profiles at two points on a fiber from an unused Olyset net, and on fibers from two depleted nets recovered from household use in rural Kenya. The fiber was sputtered with 20 nA 3keV Cs^+^ over 120 μm × 120 μm and the secondary ions were analyzed with 0.3 pA 25 keV Bi_3_^+^ after each cycle of sputtering over 50 μm × 50 μm from the center of crater area. Permethrin (PM) concentrations shown for the depleted nets were obtained using conventional gas chromatography.

Depth profiles of unimplanted Olyset fibers are shown in Fig 6. A fiber of one unused net was analyzed at two points, one with a high surface chlorine content, and a second with low surface chlorine. With increasing depth, the permethrin concentration measured at both points approached the same value of approximately 4 wt%. In both cases the surface concentration was measurably different than the concentration measured less than a micrometer into the fiber, showing surface enrichment in one case and surface depletion in the other. For comparison, fibers from two Olyset nets recovered from households in rural sub-Saharan Africa showed diminished permethrin content consistent with values obtained by conventional gas chromatographic analysis and well above the limit of detection.

If there was contamination due to handling, an increase at the surface would be expected where a depletion is actually observed. It is also known that the typical signature for a surface contaminant would be a very sharp drop in concentration as the ion beam sputters through the contaminant.

### 3D distribution of permethrin in fibers

Combining the lateral and depth resolution capabilities of ToF-SIMS makes it possible to generate three-dimensional concentration information of the species of interest. This is done by producing a two-dimensional map of the secondary ion emissions at successive sputtering levels. A 256 x 256 point scan carried out over a 50 μm x 50 μm area of a fiber at 13 sputtering levels (surface plus 12 subsurface levels to a maximum depth of 1.89 μm) produced 851968 spatially discrete values of ^35^Cl^-^ secondary ion intensity (*I*) with relative values ranging 0 to 125. The distribution of these intensities (and therefore the local Cl concentrations) closely followed a lognormal curve with the mode, median and mean of 9, 12 and 15.04, respectively.

**Fig 7:**
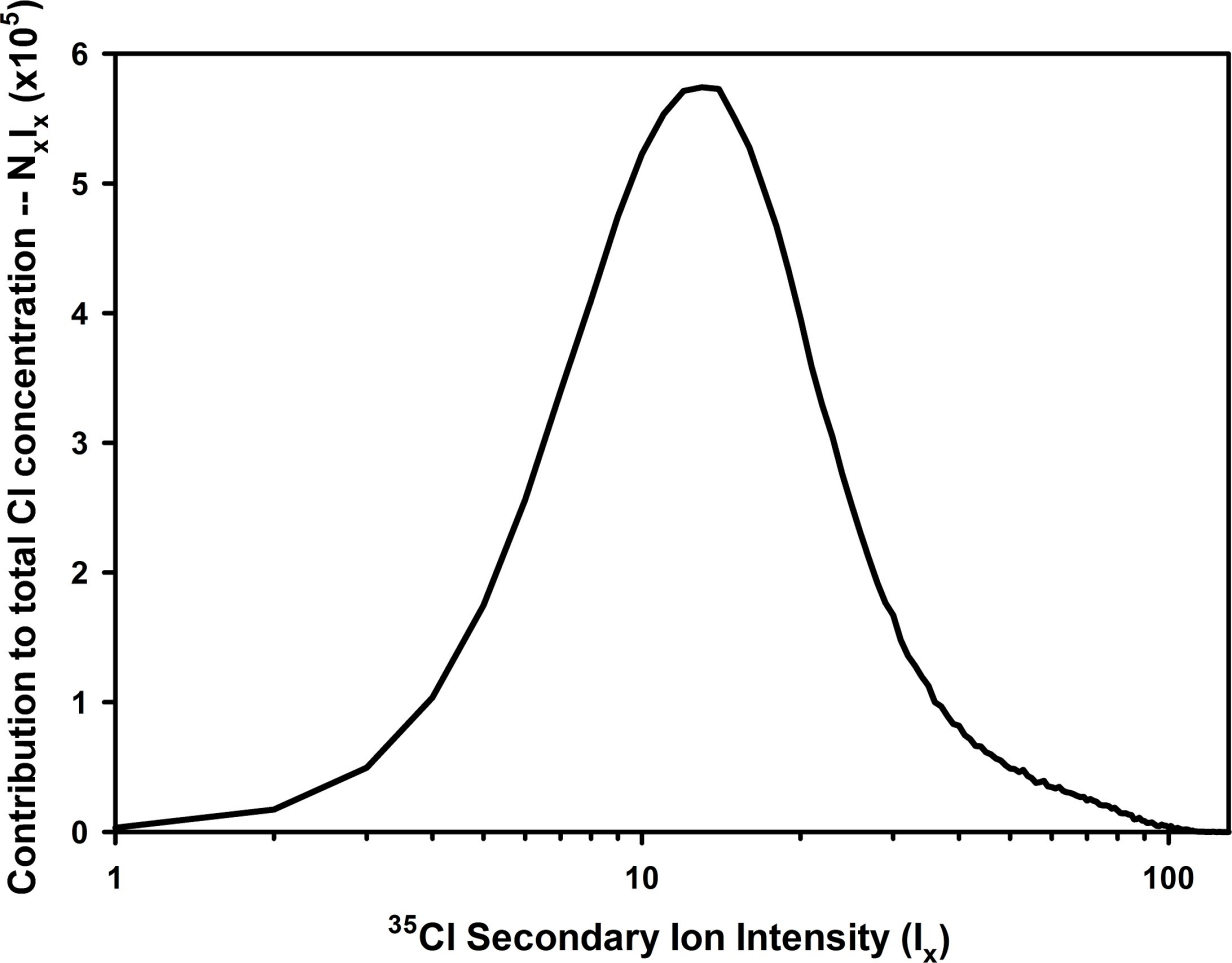
Contribution of all of the intensity regions to the overall concentration of chlorine in the sample.

**Fig 8:**
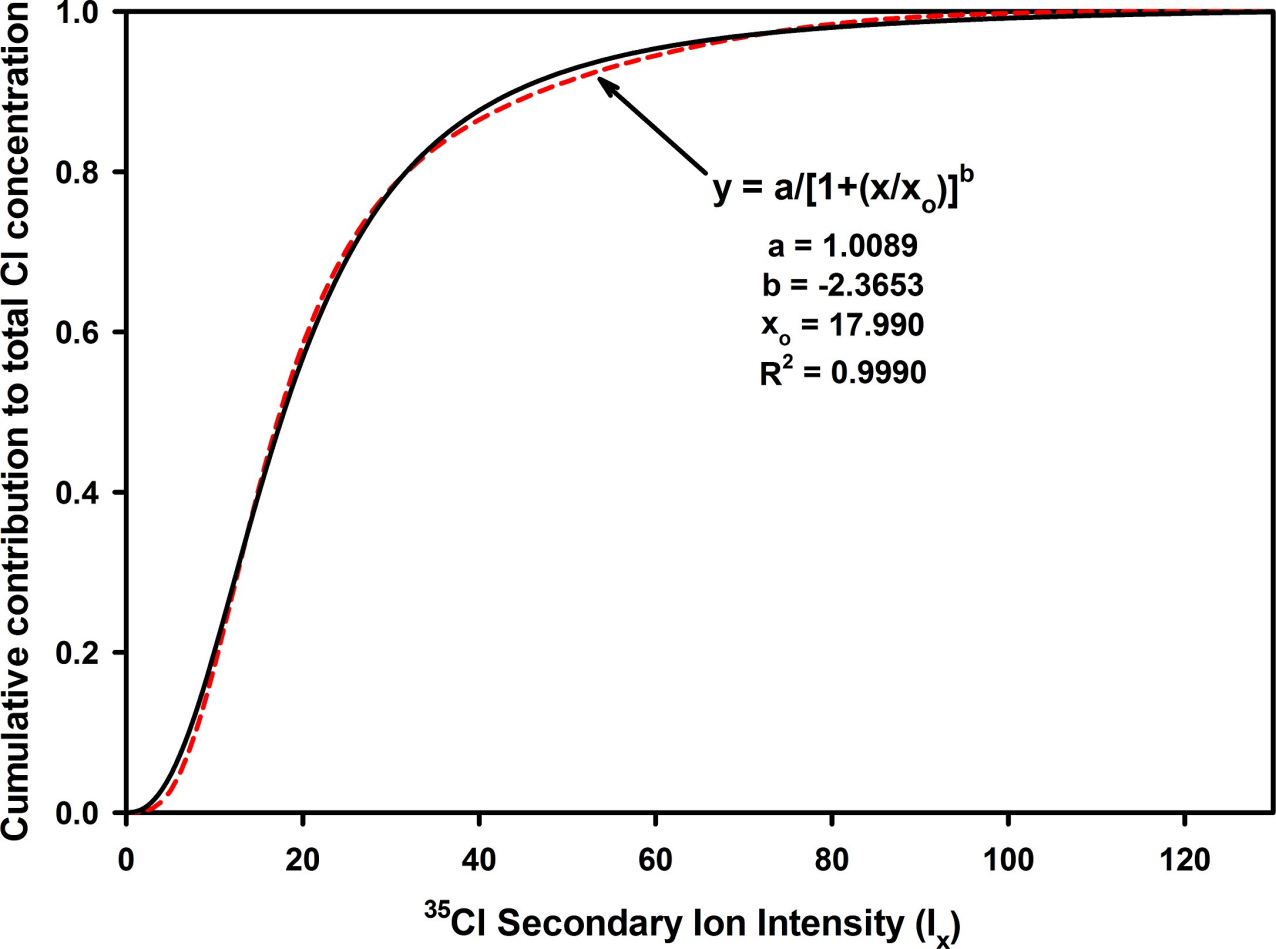
Cumulative distribution function and fit to the 3-parameter logistic curve.

With this information, it is possible to quantitatively describe the Cl concentration variance within the 50 μm x 50 μm x 1.89 μm volume of the fiber being analyzed. Recognizing that the total Cl content within this volume is

$${\left[Cl\right]}_{total}\propto I_{total}=\sum_{x=0}^{125}N_{x}I_{x}$$

the contribution of all the points at a given intensity to the overall chlorine content will be the product, N_x_I_x_, where N_x_ is the number of points at a given intensity and I_x_ is the intensity value. By plotting N_x_I_x_ against I_x_, it is possible to see the relative contribution of each of the intensity (i.e., concentration) regions to the overall concentration of Cl in the sample (Fig 7). The curve produced is log normal with a cumulative distribution function (Fig 8) closely fitting the 3-parameter logistic curve (R^2^ = 0.9990):

$$y=\frac{1.0089}{1+{\left(\frac{I_{x}}{17.990}\right)}^{-2.3693}}$$

Using this expression, it is possible to calculate that 50% of the Cl in the sample resides in regions having I_x_ ≤ 18.1. Likewise, only 3.3% of the Cl is residing in regions having I_x_ ≥ 50. Therefore, despite the presence of high concentration domains in the fiber, the large volume region of low concentration carries most of the total Cl load.

**Fig 9:**
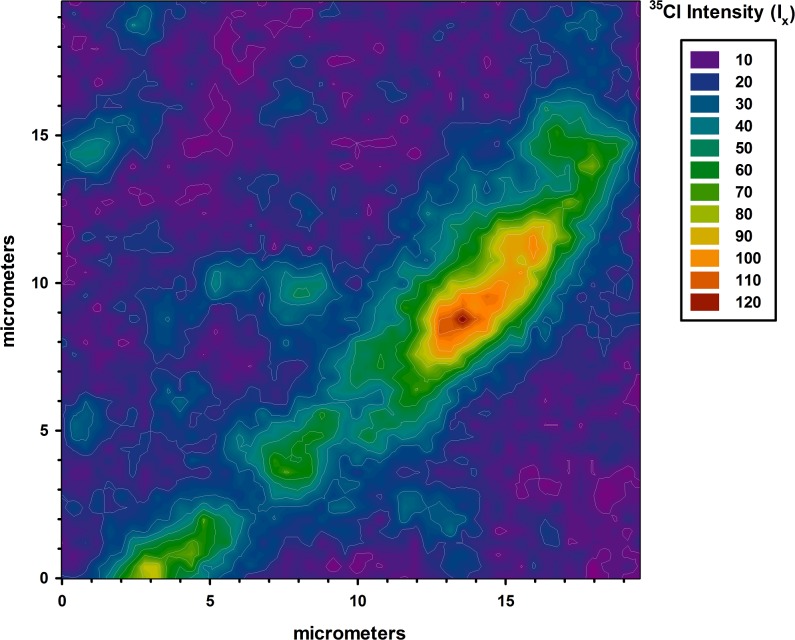
Two-dimensional map of data taken at 1.89 μm below the surface of the fiber at a location containing a high-chlorine domain.

Although they occupy a small volume fraction of the fiber, discrete domains having high ^35^Cl^-^ secondary ion intensities were found throughout. Fig 9 shows a two dimensional map of data taken at 1.89 μm below the surface of the fiber at a location containing a high-chlorine domain. In addition to showing the maximum chlorine concentration to be 12 times higher than the baseline, the contour spacing around the domain suggests that the boundary is diffuse. The molecular composition of these domains cannot be confirmed, since the Cs^+^ sputtering process destroys much molecular structure information. Nevertheless, it is likely that they either consist of permethrin that has phase-separated from the HDPE matrix, or some unidentified chlorinated additive that is poorly soluble in HDPE.

**Fig 10:**
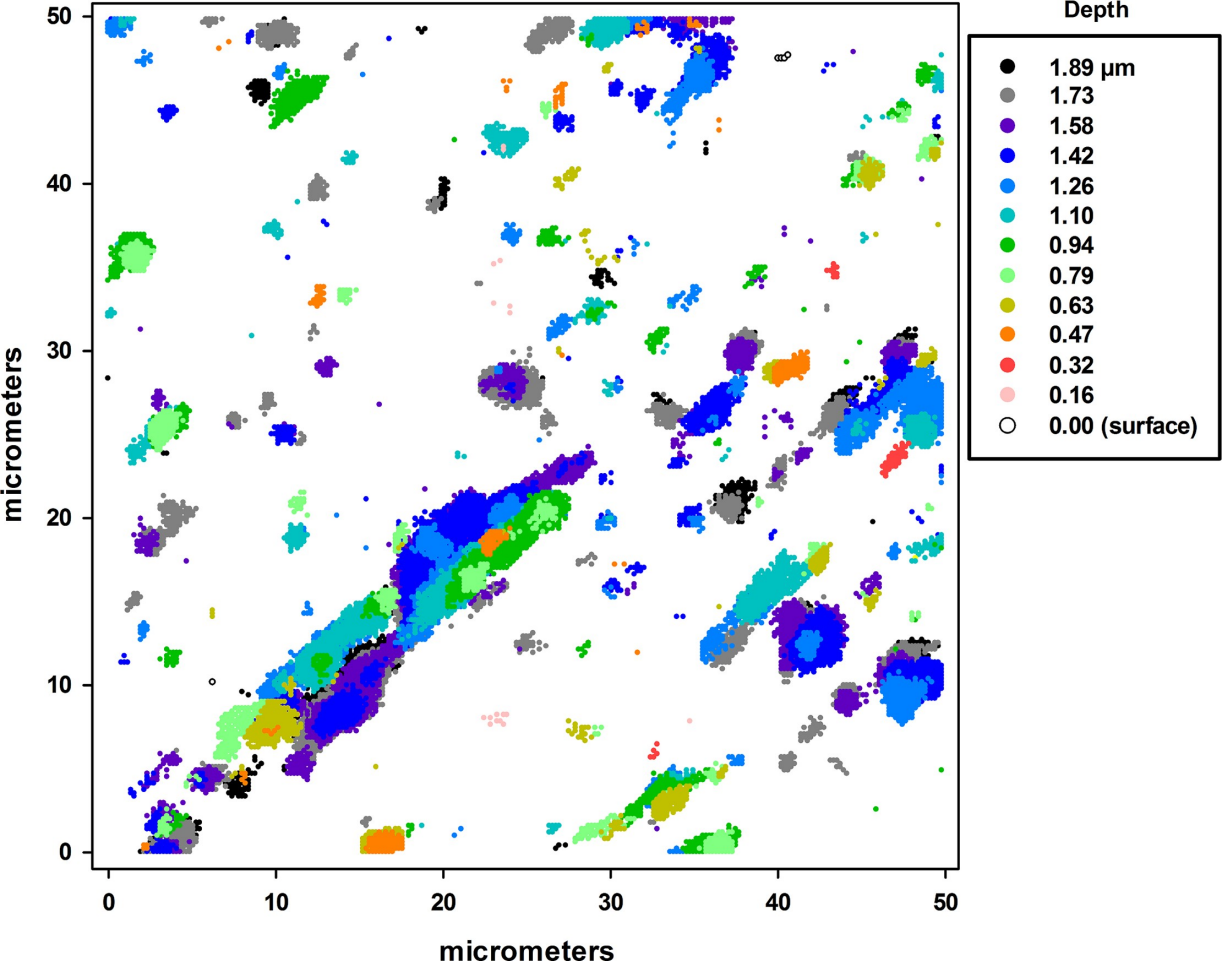
Three-dimensional stacked scatterplot of the points with high ^35^Cl^-^ secondary ion emissions (I_x_ ≥ 50) in a 50 μm x 50 μm area of an acetone-washed fiber with the depth of each data point indicated by its darkness. Data is plotted to simulate the view from the fiber surface into the direction of the core. Data points lying closest to the surface (depth < 0.5μm) are circled for clarity.

Fig 10 shows a 3D scatterplot of the locations of points with high ^35^Cl^-^ secondary ion emissions (I_x_ ≥ 50) in a 50 μm x 50 μm area of fiber with the depth of each data point indicated by its darkness. This provides depth information about the connectivity of high concentration domains. In this case, the ^35^Cl concentration near the surface is almost entirely below the I_x_ = 50 threshold and shows no connectivity with the higher concentration domains. This lack of connectivity does not imply that permethrin cannot diffuse to the surface from these presumably permethrin-rich domains, since these domains represent only 3.3% of the total chlorine contained in the sample and the balance is broadly distributed throughout the balance of the fiber volume. This figure also shows the larger domains to be strongly oriented, suggesting that they were mechanically deformed, presumably during the manufacture of the fiber. These domains also vary greatly in size, with the largest in this figure being approximately 3 μm x 20 μm.

## Discussion

Olyset nets are designed to have sufficient permethrin insecticide on the fiber surface to kill susceptible mosquitoes on contact. If the permethrin is removed by washing, handling, etc., additional permethrin is expected to spontaneously migrate from the fiber interior to the surface, restoring insecticidal performance. ToF-SIMS images from this study confirm that permethrin is present on as-received Olyset fiber surfaces, and following its removal with acetone, permethrin reappears at the surface within 4 days at 30°C. Although the return of insecticidal performance after washing has been reported in numerous publications, [4, 20–25] this is the first published instance where permethrin replenishment has been directly visualized and quantified on Olyset fiber surfaces.

### Permethrin heterogeneity at the fiber surface

Permethrin observed on the surfaces of as-received Olyset fibers was qualitatively and quantitatively different from that on fibers that were washed and incubated. At the dimensional scale of analysis, permethrin did not appear as a uniform coating, but instead as discrete, high concentration patches in both the as-received and the washed/incubated cases. This made it necessary to take measurements on many points (usually 20) of each specimen in order to ensure that a sampling was representative. This non-uniformity may be caused by the emergent permethrin moving laterally to form pools. For unwashed fibers, the RSDs of normalized ^35^Cl^-^ secondary ion intensities ranged from 32.7% to 54.7%. Although the permethrin on washed/incubated fibers appeared more diffuse than the as-received fibers, the RSDs were still quite high (18.1–45.3%). The mean level of permethrin on the surfaces of washed/incubated fibers was essentially the same for all four net samples tested, even though the initial concentrations varied significantly. In each case, the permethrin level on the surfaces of the washed/incubated fibers was lower than the initial levels, but the percent recovery varied widely because of the variation in the initial concentrations. No significant change was seen in the mean surface concentrations between 4 and 10 days of incubation, indicating that the blooming process had reached equilibrium within 4 days.

### Effect of thermal and mechanical stress

The differences seen in permethrin on the surfaces of as-received vs. washed/incubated fibers can be explained by considering the differences in thermal and mechanical stress conditions during the blooming process for each case. During fiber manufacture, permethrin is presumably fully dissolved in the polymer melt at an elevated temperature. As this melt is extruded, cooled and stretched, the polymer partially crystallizes, forcing the permethrin to concentrate in the amorphous regions of the fiber. It is during this process that a portion of the permethrin segregates to the fiber surface. The rate and extent of segregation will depend on the temperature, cooling rate and stretching forces applied, and variations in these are probably the reason for the variations in mean surface concentrations between nets as received. Mechanical stresses during knitting and product storage temperatures may also contribute to these variations. In contrast, permethrin blooming in the washed/incubated samples took place under identical, quiescent conditions at a well-controlled temperature. In this case, the extent of blooming was essentially the same from net to net, and lower than that observed before washing. An immediate consequence of this analysis is the understanding that the initial concentration of permethrin on the surface of the net is likely to bear little relevance to concentration after the first wash/recovery cycle. For this reason, quality assessment of this and similar products should probably include insecticidal potency after the first wash/blooming cycle, instead of relying on data from pristine samples.

### Diffusion of insecticide to the surface (blooming)

It was noted that after washing for 1 min in acetone, a greatly diminished, but still measurable, ^35^Cl secondary ion emission was consistently observed. Although it may be due to incomplete removal of permethrin, it may also be due to the initial emergence of permethrin from the interior. The time between washing and data collection ranged from about 2 to 4 hours, and the rate of diffusion to the surface is expected to be highest at the beginning, so this is not an unreasonable possibility, especially given the sensitivity of this technique to Cl detection. Additional experiments to measure the rate of permethrin migration to the surface would serve to clarify this.

The phenomenon of low molecular weight additive migration from the bulk to the surface of polymers has been studied for many years, largely because of its role in the premature degradation of polymers from antioxidant and photostabilizer loss [26]. “Blooming” is a special case of additive migration, whereby the additive is not continuously removed from the surface by evaporation or dissolution, but instead accumulates as a surface precipitate, as is observed in this study of Olyset fibers. Calvert and Billingham [27] suggested that blooming can only occur when the blooming species is supersaturated in the polymer, and modelled this process assuming that it continues until the concentration at the polymer surface falls to saturation. After reaching this point, further removal of additive from the surface requires evaporation or solvent leaching.

Blooming has been used to advantage in the design of certain types of monolithic controlled-release devices, in which an active ingredient (a.i.) is dispersed in a polymer well above its saturation concentration. The a.i. is present in the polymer as distinct dispersed phases embedded within a continuous phase consisting of polymer saturated with the a.i. [28, 29]. The presence of distinct phases makes this a more complex model than that of Calvert and Billingham. In this case, blooming is driven by the spontaneous migration of a.i. molecules from the disperse phase to the continuous phase, instead of the precipitation of solute from a saturated solution. In this model, the disperse phases would be expected to shrink as the a.i. accumulates on the surface, and disappear altogether when the overall a.i. concentration in the polymer falls to saturation. This process has been described as analogous to Ostwald ripening in crystalline suspensions, whereby larger crystals spontaneously grow in size at the expense of smaller crystals [26]. As with the Calvert and Billingham model, further blooming halts as soon as the a.i. reaches overall saturation in the polymer and further removal requires evaporation or leaching from the surface.

### Permthrin heterogeneity in the bulk fiber

Both two- and three-dimensional ToF-SIMS imaging confirm the existence of permethrin-rich domains of Olyset fibers, and that these domains are widely scattered and vary greatly in size. Surprisingly, these domains collectively contain only about 3 vol% of the permethrin within the fiber, based on the secondary ^35^Cl^-^ emission intensities. The remainder is diffusely distributed (dissolved) in the regions between these domains. However, since only one site on a fiber was examined, this result may not be representative of the Olyset nets in general.

Quantitative depth profile analysis also confirmed that the concentration of permethrin in the bulk fiber is not a reliable indicator of the concentration at the surface. Concentration in the fiber subsurface (ca. 1 to 5 μm from the surface) was found to be mostly uniform with respect to depth, but in some cases deviated strongly between 1 μm and the surface. The quantitative depth profiling method described in this article was capable of determining permethrin concentration to a detection limit of 0.051wt%.

## Conclusions

ToF-SIMS analysis has been demonstrated as a useful method for imaging and quantifying permethrin insecticide on the surface and in the interior of Olyset fibers. Using the ion-implantation for calibration, quantitative depth profiling was conducted with a limit of detection of 0.051 wt% permethrin. In some cases, the concentrations of permethrin within the first micrometer of the fiber surface were found to be significantly different than in the bulk.

The presentation of permethrin on the fiber surface was found to be qualitatively and quantitatively different for as-received fibers versus fibers that have been acetone-washed and incubated. In both cases, permethrin deposits were patchy, but somewhat more diffuse in appearance after washing/incubation. Permethrin levels on washed/incubated samples were consistently lower than as-received samples. These observations are likely due to the different set of conditions under which permethrin blooming occurred during melt spinning versus quiescent incubation. For this reason, it is recommended that the quality of insecticidal performance be assessed not only for freshly made nets, but also samples that have been washed and incubated, since surface permethrin levels may be significantly different in the two cases.

Two- and three-dimensional imaging suggest that permethrin is dissolved throughout the fiber, but also small domains of highly concentrated permethrin are dispersed throughout. In the fiber section studied, these domains collectively occupied only about 3% of the fiber volume. It is expected, based on models of monolithic controlled-release devices, that as permethrin is lost from the fiber, these domains will decrease in size. Once these domains disappear altogether, the driving force for blooming will cease, and the insecticidal power of the fiber will be lost, even if the fiber still contains significant insecticide. This underscores the need for quality assessment methods beyond the measurement of total insecticide in the net.

Although the present study focused on the Olyset permethrin/HDPE system, it is expected that other insecticide-incorporated LLINs can be evaluated similarly. Furthermore, as mosquitoes in malaria-endemic areas develop resistance to pyrethroid insecticides,[30] nets having new active ingredients, e.g. piperonyl butoxide [31], are being considered. A deeper understanding of the release characteristics of these products is essential for their development and testing if they are to contribute to the continued reduction of the malaria burden worldwide. This work demonstrates how ToF-SIMS analysis can be a powerful tool to further this effort.
